# A Case of Tubercular Disease

**Published:** 1844-05

**Authors:** W. W. Goodwin

**Affiliations:** Utica, Indiana


Art. II.—*A Case of Tubercular Disease.* By W. W. Goodwin, M.D., of Utica, Indiana.

November 24, 1843, I was called to visit Mrs. B., æt. about 30 years, of scrofulous diathesis, who had been attacked some ten months previously with slight diarrhœa, attended with some cough, and pain in the chest, from all of which symptoms she at first had occasional respites, continuing for some weeks. After remaining some time in the above condition, she became pregnant with her fifth child, when all the pectoral symptoms grew much less conspicuous, while the diarrhœa gradually increased in the frequency of its attacks until I saw her. At this time she was laboring under a regular tertian ague. She was much emaciated, confined to her bed, pulse rather tense, and 130 in the minute; tongue furred in the centre, with moist, clean edges and tip. Tenderness to pressure in epigastrium and region of the colon. The sound elicited by percussion over the inferior lobe of the left lung very slightly cavernous—that from the superior lobe rather dull. Right lung rather dull throughout, but the departure from a healthy sound was not great. As the disease advanced, the sound on percussion underwent some modification, becoming more healthy, until her accouchement took place. Her labour was somewhat tedious, but in other respects was not unnatural. As is usual in such cases, after this event, she grew worse, her cough becoming very troublesome, attended with severe pain in the left side, which soon extended to the right also. Purulent expectoration came on, and was followed in a few days by considerable hemoptysis, after which she declined rapidly. Pain in the chest, stomach and bowels increased to an extent that was excruciating; sweating and diarrhœa became profuse; pulse small, and from 130 to 150. Sweating ceased a few days before death. The sound on percussion over the inferior lobe of the left lung very hollow—over other parts rather dull.

An autopsy was made about five hours after the death of the patient.

*Chest.*—Left lung very much reduced in volume, with several considerable cavities in its inferior lobe. The balance a complete mass of tubercles in different degrees of maturation—extensive adhesions of the superior posterior surface. Right lung as extensively diseased as the left, but the tubercles not so far advanced; the adhesions not quite so great. Heart and pericardium normal.

*Abdomen.*—Liver gorged with dark blood; gall-bladder containing dark viscid bile. Spleen, pancreas, and kidneys natural. Stomach had a small ulcer near its cardiac orifice; duodenum healthy; other small intestines ulcerated in many places, adhering strongly to the abdominal parietes. Colon ulcerated in several places, and very much thickened and contracted in volume, particularly the ascending portion of the bowel. Mesentery softened, and broken down in places; glands greatly enlarged.

No examination was made of other parts, the character of the disease having been fully revealed by what was inspected. I may remark that the treatment, which would not be interesting in detail, was directed to the relief of symptoms, the case being one in which, from the beginning, but slender hope of recovery was afforded.

March 15, 1844.

